# Quercitrin, an Inhibitor of Sortase A, Interferes with the Adhesion of *Staphylococcal aureus*

**DOI:** 10.3390/molecules20046533

**Published:** 2015-04-13

**Authors:** Bingrun Liu, Fuguang Chen, Chongwei Bi, Lin Wang, Xiaobo Zhong, Hongjun Cai, Xuming Deng, Xiaodi Niu, Dacheng Wang

**Affiliations:** 1 College of Animal Science, Jilin University, Changchun 130062, China; E-Mails: lhglbr@outlook.com (B.L.); chenfuguang@caas.cn (F.C.); bicwei@163.com (C.B.); 15043029208@163.com (X.Z.); chj6788571@163.com (H.C.); 2 State Key Laboratory of Veterinary Biotechnology, Harbin Veterinary Research Institute of Chinese Academy of Agricultural Science, Harbin 150001, China; 3 Key Laboratory of Zoonosis Research, Ministry of Education, Institute of Zoonosis, College of Veterinary Medicine, Jilin University, Changchun 130062, China; E-Mails: chj6788571@163.com (L.W.); dengxm@jlu.edu.cn (X.D.); 4 Department of Food Quality and Safety, Jilin University, Changchun130062, China

**Keywords:** sortase A, quercitrin, *Staphylococcus aureus*, molecular modeling

## Abstract

Sortase A (SrtA) is a cysteine transpeptidase of most Gram-positive bacteria that is responsible for the anchorage of many surface protein virulence factors to the cell wall layer. SrtA mutants are unable to display surface proteins and are defective in the establishment of infections without affecting microbial viability. In this study, we report that quercitrin (QEN), a natural compound that does not affect *Staphylococcus aureus* growth, can inhibit the catalytic activity of SrtA in fibrinogen (Fg) cell-clumping and immobilized fibronectin (Fn) adhesion assays. Molecular dynamics simulations and mutagenesis assays suggest that QEN binds to the binding sites of the SrtA G167A and V193A mutants. These findings indicate that QEN is a potential lead compound for the development of new anti-virulence agents against *S. aureus* infections.

## 1. Introduction

The Gram-positive pathogenic bacteria, *Staphylococcus aureus* (*S. aureus*), is the most commonly isolated human bacterial pathogen, which persistently and asymptomatically colonizes up to 20%–30% of humans and intermittently colonizes up to 50%–60% [1]. *S. aureus* colonizes human skin and mucosa, it also can cause many infectious diseases ranging from minor skin infections and abscesses to life-threatening diseases (such as necrotizing pneumonia, endocarditis, and septicemia) associated with high morbidity and mortality [2]. Methicillin-resistant *S. aureus* (MRSA) was first reported only two years after methicillin was applied for the treatment of penicillin-resistant bacteria. The reduced efficacy of vancomycin and linezolid against MRSA makes research into developing new treatment options a priority. 

The sortase enzymes, produced by most of Gram-positive bacteria, are a family of transpeptidases which can mediate the anchorage of surface protein virulence factors to the peptidoglycan cell wall layer via a cell C-terminal sorting signal reaction [3]. Numerous previous studies using knockout experiments have demonstrated that the sortase A (SrtA) isoform is constitutively expressed and cleaves the amide bond between the threonine and glycine of the LPXTG motif, which is found in many surface proteins that fulfill diverse functions during the infection process [4]. These surface proteins, used by Gram-positive pathogenic bacteria, play a critical role in the many processes of the establishment of an infection, including adherence, colonization, invasion, signaling and evasion of the host immune system [5]. Mazmanian *et al.* have evaluated the potential role of surface proteins in the pathogenesis of *S. arueus* infections using the mutant that lack the *srtA* gene [6]. Knockout mutations of the *srtA* gene in *S. aureus* interfere with the assembly of surface proteins into their envelope, preventing abscess formation in organ tissues or causing lethal bacteremia when inoculated into the bloodstream of mice [7,8]. *Streptococcus gordonii SrtA* mutants also failed to display the surface proteins, leading to a significant decrease of bacterial adhesion [9]. Therefore, SrtA inhibitors represent promising candidates for the development of therapeutics for the Gram-positive bacterial infections and may lead to new antibacterial drugs with novel mechanisms of action [10,11,12,13].

Currently, the increasing disease burden and the declining performance of traditional antimicrobial agents to combat bacteria-mediated disease make the development of alternative therapeutic strategies priority. Anti-virulence strategies have become a hot topic. We have screened anti-SrtA molecules from a number of detoxifying natural compounds by measuring their rate of inhibition of SrtA enzymatic activity. Quercitrin (QEN) was selected for to its strong inhibitory effect in our assays. In the present study, we observed that QEN, a natural bioflavonoid from *Sabina pingii var. wilsonii* that does not interfere with bacterial growth, could inhibit the catalytic activity of SrtA by binding directly to the active region of the SrtA, indicating that this agent may be a useful lead compound for developing novel antiinfective drugs.

## 2. Results and Discussion

### 2.1. QEN Blocks the Thioesterification Process of Sortase A Catalysis

**Figure 1:**
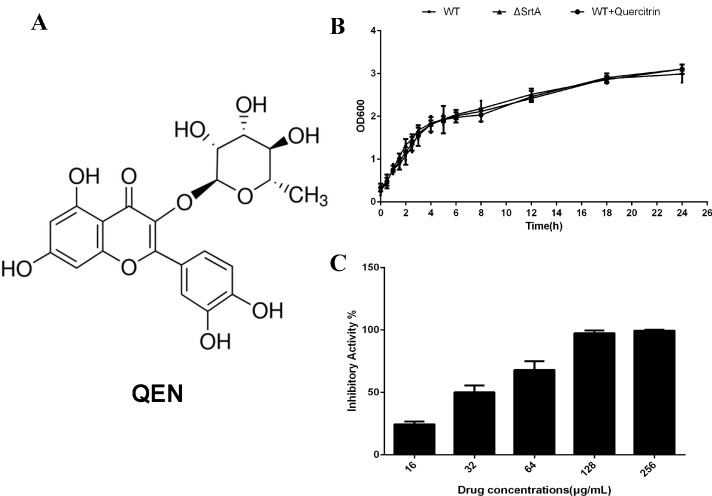
QEN blocks the thioesterification process of SrtA catalysis, but does not inhibit bacterial physiology. (**A**) The chemical structure of QEN. (**B**) The growth curve of *S. aureus* treated with or without QEN. *S. aureus* were cultured in BHI without QEN or with 256 μg/mL QEN, and the absorbency readings of the samples were taken at OD_600_ at the indicated time point. (**C**) QEN blocks the catalytic activity of SrtA. The fluorescent peptide was incubated with purified SrtA treated with the indicated concentrations of QEN at 37 °C for 30 min, and the fluorescence was measured 1 h after adding the substrate-modified peptide.

Among the 27 compounds screened (Table 1), we found that QEN, a natural bioflavonoid from *Sabina pingii var. wilsonii*, was able to remarkably inhibit the catalytic activity of purified SrtA via sortase activity inhibition assay. In our experimental system, the concentration of QEN required for 50% inhibition (half maximal inhibitory concentration, IC_50_) was 32.18 ± 5.36 μg/mL. Additionally, the minimum inhibitory concentrations (MICs) of QEN against the tested *S. aureus* strains (*S. aureus* Newman D2C and the *srtA* mutant) were both greater than 1024 μg/mL. The growth curve of Newman D2C treated with or without QEN was also monitored and showed that bacterial growth was mostly unaffected by QEN, even at a concentration of 256 μg/mL. The growth rate of the Newman ∆*SrtA* strain was similar to that of the wild-type Newman D2C strain. Taken together, our results indicate that QEN is capable of effectively inhibiting the catalytic activity of SrtA, but has little effect on bacterial growth (Figure 1B,C).

**Table 1:** Compounds screened.

Compounds		
Quercitrin	Vitexin	Isoliquiritin
Liquiritigenin	Tetrandrine	Honokiol
Cyrtopterinetin	Fisetin	Eleutheroside A
Chrysin	Daidzin	Quinine
Formononetin	Emodin	Sodium houttufonate
Tectorigenin	Psoralen	Dryocrassin
Viola yedoensis extract	Andrographolide	Wogonoside
Prunella vulgaris leaf extract	Artemisinin	Radix platycodi extract
Forsythin	Kaempferol	Berberine

### 2.2. QEN Inhibits Adherence of S. aureus to Cell-Matrix Proteins

**Figure 2:**
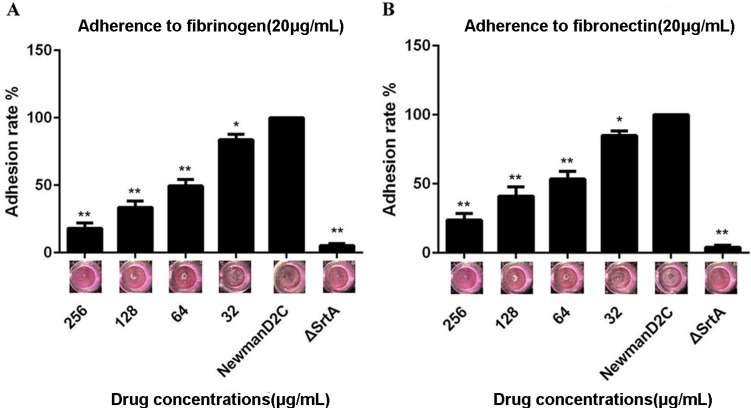
QEN inhibits the adherence of *S. aureus* to fibrinogen and fibronectin. Bacteria treated with the indicated concentrations of QEN were incubated in fibrinogen- or fibronectin-coated 96-well plates for 2 h at 37 °C, and the adhesion of *S. aureus* to fibrinogen (**A**) and fibronectin (**B**) was measured. *p < 0.05 and **p < 0.01.

An active sortase enzyme is indispensable for the adherence of *S. aureus* to host cell matrices and establishment of an infection. ∆*srtA* mutant strains cannot bind to cell-matrix proteins (such as fibrinogen and fibronectin) *in vitro* [8]. Therefore, the ability of QEN to inhibit the adherence of *S. aureus* to fibrinogen or fibronectin was tested using an assay of cell adhesion to fibrinogen-coated or fibronectin-coated plates by measuring the absorbance following staining with crystal violet. As expected, both the fibrinogen and fibronectin binding activities of the ∆*srtA* mutant strain were almost completely lost. The treatment of Newman D2C with QEN led to a significant reduce for the attachment of bacteria to fibrinogen-coated or fibronectin-coated surfaces. Importantly, when treated with 64 μg/mL QEN, the absorbance values (OD at 570 nm) were only 51.90% and 49.60% in the fibrinogen binding assay and the fibronectin binding assay (Figure 2A,B), respectively. Taken together, our results suggest that QEN inhibits SrtA activity *in vitro* and in turn reduces fibrinogen-binding protein and fibronectin-binding protein surface display.

### 2.3. Determination of the Binding Mode of SrtA with QEN

**Figure 3:**
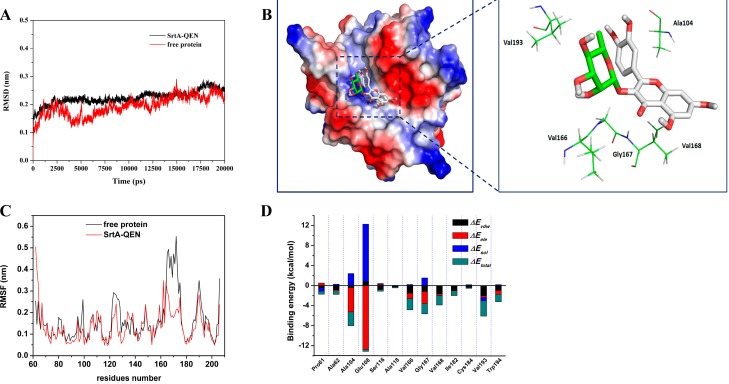
Structures of the screening compounds. (**A**) The root-mean-square deviations (RMSD) of all the atoms of SrtA-QEN complexes with respect to their initial structures as a function of time. (**B**) The predicted binding mode of QEN in the SrtA binding pocket obtained from the MD simulation. (**C**) RMSFs of residues of the whole protein in the SrtA-QEN complex and free SrtA during the last 10-ns simulation. (**D**) Decomposition of the binding energy on a per-residue basis in the SrtA-QEN complex.

Results from our functional analyses strongly suggested direct binding of SrtA by QEN; we next explored the mechanism of action of this agent by studying its interactions with the enzyme using molecular modeling. The initial structure of SrtA was obtained from the NMR structure (PDB code: 1IJA) [14]. The preferential binding mode of SrtA with QEN was determined by 20-ns molecular dynamics simulations based on the docking results. The root-mean-square deviation (RMSD) values of the protein were calculated and are plotted in Figure 3A. As shown in Figure 3A the protein structures of all the systems were stabilized during the simulations. 

In the simulation, QEN can bind to SrtA via hydrogen bonding and hydrophobic interactions as a ligand. Over the time course of the simulation, QEN localizes to the active region, which is reported to participate in reactivity and is important for SrtA function [15]. The structure of SrtA-QEN is illustrated in Figure 3B, and the electrostatic potentials of the protein were mapped using the APBS software. Specifically, the structure of SrtA-QEN (Figure 3B) showed that the 4*H*-chromen-4-one moiety of QEN formed both a strong interaction with the side-chain amino group of Ala104 and the side-chain carbonyl group of Ser106. Moreover, the side chains of Val193, Val166, Gly167, and Val168 can form van der Waals interactions with QEN (Figure 3A

The root mean square fluctuations (RMSF) of the residues of the whole protein in the SrtA-QEN complex and free protein were calculated, as shown in Figure 3C which clearly depicts the different flexibilities in the binding site of SrtA in the presence and absence of QEN. All of the residues in the SrtA binding site that bind to QEN show a RMSFs of less than 4.00 Å, with a smaller degree of flexibility, compared with those of free SrtA, which is indicated that these residues seem to be more rigid due to the binding to QEN. These results indicate that the stabilization at the binding sites of SrtA in this complex was mostly due to residues Ala104, Val193, Val166, Gly167, and Val168, as shown in Figure 3B. 

### 2.4. Identification of the Binding Site in the SrtA-QEN Complex

The energy contributions from the selected residues are summarized in Figure 3D. Ala104 had an appreciable electrostatic (ΔE_ele_) contribution, with a ΔE_ele_ of ≤−5.0 kcal/mol (Figure 3D). In fact, Ala104 is close to the 4*H*-chromen-4-one moiety of QEN, and electrostatic interactions exist, leading to one strong electrostatic interaction between SrtA and QEN. In addition, the Val166, Gly167 and Val168 residues, with ΔE_vdw_’s of ≤ −2.0 kcal/mol, have strong Van der Waals interactions with QEN due to the close proximity between these residues and QEN. Moreover, the Val193 residue, with a ΔE_vdw_ of ≤ −3.0 kcal/mol, also has a strong Van der Waals interaction with QEN. Except for the Val168 and Val193 residues, the majority of the binding free energy originated from electrostatic interactions. The total binding free energy for the SrtA-QEN complex and its detailed energy contributions, are summarized in Table 2. With the summation of the solute entropy term (~5.9 kcal/mol), an estimated ΔG_bind_ of −11.7 kcal/mol was calculated for QEN, suggesting that QEN can strongly bind to the activity cavity of SrtA. 

**Table 2:** The calculated energy components, binding free energy (kcal/mol), binding constants and number of binding sites of the WT-QEN, G167A-QEN and V193A-QEN systems based on the fluorescence spectroscopy quenching method.

	TΔ*S* (kcal/mol)	Δ*G_bind_* (kcal/mol)	Binding Constants *K*_A_ (1 × 10^5^) L·mol^−1^	n
WT-QEN	5.9 ± 1.8	−11.7 ± 1.4	6.9 ± 1.3	0.9991
G167A-QEN	6.2 ± 1.6	−5.6 ± 1.9	3.7 ± 0.7	1.0044
V193A-QEN	6.1 ± 1.5	−7.4 ± 1.3	4.2 ± 1.1	0.9996

To examine the accuracy of the binding site in the complex, the mutant complexes G167A-QEN and V193A-QEN were used as the preliminary structures for MD simulations. The MM-GBSA calculation predicted that G167A and V193A bind more weakly to QEN than the WT-SrtA (−5.6 kcal/mol for G167A and −7.4 kcal/mol for V193A), as shown in Table 2. The calculations for G167A and V193A showed that these mutations resulted in a decrease of approximately 7 kcal/mol of binding energy compared with the WT-SrtA. According to the experimental results, the binding constants or KAs of the interaction between QEN and SrtA decreased in the following order: WT > V193A > G167A. This result indicates that WT-SrtA has the strongest ability to bind to QEN and G167A has the weakest ability, as shown in Table 2. The calculated binding free energies were consistent with the experimental data. Therefore, MD simulations exactly generated a reliable SrtA-QEN complex. 

The Ala104, Val193, Val166, Gly167, and Val168 residues have a key role in the binding of QEN to SrtA, which is identical with previous reports [15,16]. It is plausible that due to the binding of QEN with the activity region (the Ala104, Val193, Val166, Gly167, and Val168 residues), the biological activity of SrtA was inhibited. 

### 2.5. Discussion

Compared with traditional strategies that are aimed at killing bacteria or preventing their growth, this anti-virulence approach diminishes the rate of development of bacterial resistance. Furthermore, the specific effect of anti-virulence drugs could aid the host immune system in preventing bacterial colonization or clearing established infections [5,17,18,19]. In this study, we demonstrated that QEN, a natural bioflavonoid from Sabina pingii var. wilsonii without any effect on bacterial growth, was able to significantly inhibit the catalytic activity of SrtA, a critical virulence factor that mediates the anchorage of surface protein virulence factors to the peptidoglycan cell wall layer of Gram-positive pathogens. Importantly, the binding activity of *S. aureus* to fibrinogen or fibronectin was remarkably reduced upon treatment with QEN compared with untreated controls, suggesting that QEN is an effective inhibitor of SrtA activity *in vivo*. In summary, the above findings indicate that QEN can inhibit the catalytic activity of SrtA *in vitro* without anti-bacterial activity and may be used as a promising lead compound for the development of an anti-virulence agent against *S. aureus* infections specially targeting SrtA. Additionally, the data presented in this work further provides the basis for analysis of the structure-activity relationship and *in vitro* activity of sortase A inhibitors.

## 3. Experimental Section

### 3.1. Bacterial Strains, Plasmids and Reagents

The bacterial strains and plasmids used in this work are presented in Table 3. *S. aureus* Newman D2C and its *srtA* mutant were cultured in brain-heart infusion (BHI) media at 37 °C. The fluorescent peptide Dabcyl-QALPETGEE-Edans was synthesized by GL Biochem (Shanghai, China). Compound quercitrin (QEN) was purchased from the National Institutes for Food and Drug Control of Chain and dissolved in DMSO (Sigma-Aldrich, St. Louis, MO, USA). Other reagents were obtained from Sigma-Aldrich unless otherwise noted.

**Table 3:** Strain and plasmid list.

Strain or Plasmid	Relevant Genotype	Source or Reference
**Strains**		
***S. aureus***		
Newman D2C	Wild-type SrtA positive; nonhemolytic; coagulase Negative	ATCC25904
ΔSrtA	srtA::Em^r^; isogenic mutant of Newman D2C	
***E. coli***		
BL21	Expression strain, F^−^ *ompT hsdS(r_B_^−^ m_B_^−^) gal dcm* (DE3)	Invitrogen
**Plasmids**		
pGEX-6P-1	Expression vector	Amersham
pGSrtA_ΔN59_	pGEX-6P-1 with *srtA* gene	This study
G167A	pGSrtA_ΔN59_ derivative, for the substitution of Gly167 with alanine	This study
V193A	pGSrtA_ΔN59_ derivative, for the substitution of Val192 with alanine	This study

### 3.2. Preparation of Recombinant SrtA_ΔN59_ and Its Mutant

The DNA fragment encoding SrtA_ΔN59_ (residues 60-206) was PCR-amplified from the genomic DNA of *S. aureus* Newman D2C using the primers PsrtA59F and PsrtA59R and cloned into the pGEX-6P-1 vector to generate the pGSrtA_ΔN59_ construct. Site-directed mutagenesis was performed on pGSrtA_ΔN59_ to produce the mutations G167A and V193A using the QuikChange Site-Directed Mutagenesis Kit according to the manufacturer’s protocol (Stratagene, La Jolla, CA, USA). The pGSrtA_ΔN59_ plasmid and the mutant constructs were transformed into BL21 Escherichia coli (Invitrogen, Carlsbad, CA, USA). The transformed bacteria were then grown in 1 L of LB broth containing ampicillin (100 μg/mL) at 37 °C until the OD_600_ reached 0.6–0.8, and the expression of interest protein was induced with 1 mM isopropyl β-d-1-thiogalactopyranoside (IPTG, Invitrogen). The cells were harvested (5000× *g* for 5 min) after overnight culturing at 23 °C, and the recombinant protein was purified on a GST affinity column (2 mL glutathione Sepharose 4B) (GE Amersham Biosciences, Piscataway, NJ, USA). All expression vectors were confirmed via DNA sequencing. The complementary forward and reverse primer pairs used in SrtA_ΔN59_ variant construction are listed in Table 4.

**Table 4:** Oligonucleotide primers used in this study.

Primer Name	Oligonucleotide (5–3) *ᵅ*
PsrtA59F	GCGGGATCCCCGGAATTCCAAGCTAAACCTCAAATTCC
PsrtA59R	CCGCTCGAGTTATTTGACTTCTGTAGCTACAA
G167A–F	AGCCAACAGATGTAGCAGTTCTAGAT
G167A–R	AGAACGCTACATCTGTTGGCTTAACATCTC
V193A–F	TGAAAAGACAGGCGCTTGGGAAAAAC
V193A–R	TTCCCAGCGCCTGTCTTTTCATTGTAATCAT

^a^ Restriction endonuclease recognition sites or mutated codons are underlined.

### 3.3. Sortase Activity Inhibition Assay

The inhibitory activities of QEN against Sortase were determined using a fluorescence resonance energy transfer (FRET) assay, which was performed in 300 μL reactions containing 50 mM Tris-HCl; 5 mM CaCl_2_; 150 mM NaCl, pH7.5; 4 μM of recombinant SrtA_ΔN59_; and 10 μM of fluorescent peptide (Dabcyl-QALPETGEE-Edans). QEN was added to the 96-well plate to reach the indicated concentrations, and they were then incubated at 37 °C for 30 min before adding the substrate-modified peptide and allowing the reaction to proceed for 1 h at 37 °C. The fluorescence of each sample was measured with the emission and excitation wavelengths set at 495 and 350 nm, respectively. Each concentration was repeated three times independently in this experiment.

### 3.4. Determination of Minimum Inhibitory Concentration (MIC) and Growth Curves

The MICs of QEN against *S. aureus* was determined as previously described [20]. To plot growth curves, 1 mL of an overnight *S. aureus* bacteria culture was added to 50 mL of fresh BHI broth and incubated at 37 °C with or without the tested compound for 0.5, 1, 1.5, 2, 2.5, 3, 4, 5, 6, 8, 12, 18, and 24 h. Absorbency readings were taken at OD_600_.

### 3.5. Fibrinogen-Binding and Fibronectin-Binding Assays

The *S. aureus* wild-type strain was cultured overnight, diluted 1:100 in BHI broth, and incubated with the indicated concentrations of QEN in a shaking incubator at 37 °C to an initial OD_600_ of 0.05. The Newman ∆SrtA strain was grown under the same conditions and used as a positive control. All samples were cultivated for 2 h on a rotary shaker at 37 °C, and the cells were then pelleted by centrifugation (5000× *g* for 5 min), washed twice, and resuspended in PBS to an OD_600_ of 1.0 prior to their use in experiments. The cell suspensions were added to a polystyrene Costar 96-well plate, which had been coated overnight at 4 °C with 100 μL of a 20 μg/mL bovine fibrinogen (Fg) or a 20 μg/mL human plasma fibronectin (Fn) solution. After a 2 h incubation at 37 °C, the suspension was removed and the plate was stained with crystal violet for 15 min and the absorbances of the plated samples were subsequently read at 570 nm with a microplate reader. 

### 3.6. Computational Chemistry

The relevant molecular modeling methods of protein and ligands are performed based on our previously reported work [21].

### 3.7. Binding Affinity Determination of QEN with WT-SrtA_ΔN59_, G167A and V193A

The binding constants (KAs) of QEN to the binding sites on WT-SrtA_ΔN59_ (WT-SrtA), the G167A mutant, and the V193A mutants were measured using the fluorescence-quenching method, as presented in the previous report [21]. 

### 3.8. Statistical Analysis

The statistical significances of all assays were calculated using the 2-tailed Student’s *t*-test. Differences were considered statistically significant when *p* < 0.05.

## References

[1] Wertheim H.F., Melles D.C., Vos M.C., van Leeuwen W., van Belkum A., Verbrugh H.A., Nouwen J.L. (2005). The role of nasal carriage in *Staphylococcus aureus* infections. Lancet Infect. Dis..

[2] Lowy F.D. (1998). *Staphylococcus aureus* infections. N. Engl. J. Med..

[3] Oh K.B., Oh M.N., Kim J.G., Shin D.S., Shin J. (2006). Inhibition of sortase-mediated *Staphylococcus aureus* adhesion to fibronectin via fibronectin-binding protein by sortase inhibitors. Appl. Microbiol. Biotechnol..

[4] Ilangovan U., Ton-That H., Iwahara J., Schneewind O., Clubb R.T. (2001). Structure of sortase, the transpeptidase that anchors proteins to the cell wall of *Staphylococcus aureus*. Proc. Natl. Acad. Sci. USA.

[5] Cossart P., Jonquieres R. (2000). Sortase, a universal target for therapeutic agents against gram-positive bacteria?. Proc. Natl. Acad. Sci. USA.

[6] Jonsson I.M., Mazmanian S.K., Schneewind O., Verdrengh M., Bremell T., Tarkowski A. (2002). On the role of *Staphylococcus aureus* sortase and sortase-catalyzed surface protein anchoring in murine septic arthritis. J. Infect. Dis..

[7] Cheng A.G., Kim H.K., Burts M.L., Krausz T., Schneewind O., Missiakas D.M. (2009). Genetic requirements for *Staphylococcus aureus* abscess formation and persistence in host tissues. FASEB J..

[8] McAdow M., Kim H.K., Dedent A.C., Hendrickx A.P., Schneewind O., Missiakas D.M. (2011). Preventing *Staphylococcus aureus* sepsis through the inhibition of its agglutination in blood. PLoS Pathogens.

[9] Bolken T.C., Franke C.A., Zeller G.O., Hruby D.E. (2001). Identification of an intragenic integration site for foreign gene expression in recombinant *Streptococcus gordonii* strains. Appl. Microbiol. Biotechnol..

[10] Chan A.H., Wereszczynski J., Amer B.R., Yi S.W., Jung M.E., McCammon J.A., Clubb R.T. (2013). Discovery of *Staphylococcus aureus* sortase A inhibitors using virtual screening and the relaxed complex scheme. Chem. Biol. Drug Des..

[11] Zhulenkovs D., Rudevica Z., Jaudzems K., Turks M., Leonchiks A. (2014). Discovery and structure-activity relationship studies of irreversible benzisothiazolinone-based inhibitors against *Staphylococcus aureus* sortase A transpeptidase. Bioorganic Med. Chem..

[12] Kahlon A.K., Negi A.S., Kumari R., Srivastava K.K., Kumar S., Darokar M.P., Sharma A. (2014). Identification of 1-chloro-2-formyl indenes and tetralenes as novel antistaphylococcal agents exhibiting sortase A inhibition. Appl. Microbiol. Biotechnol..

[13] Uddin R., Lodhi M.U., Ul-Haq Z. (2012). Combined pharmacophore and 3D-QSAR study on a series of *Staphylococcus aureus* Sortase A inhibitors. Chem. Biol. Drug Des..

[14] Suree N., Liew C.K., Villareal V.A., Thieu W., Fadeev E.A., Clemens J.J., Jung M.E., Clubb R.T. (2009). The structure of the *Staphylococcus aureus* sortase-substrate complex reveals how the universally conserved LPXTG sorting signal is recognized. J. Biol. Chem..

[15] Ton-That H., Mazmanian S.K., Faull K.F., Schneewind O. (2000). Anchoring of surface proteins to the cell wall of *Staphylococcus aureus*—Sortase catalyzed *in vitro* transpeptidation reaction using LPXTG peptide and NH2-Gly(3) substrates. J. Biol. Chem..

[16] Ton-That H., Liu G., Mazmanian S.K., Faull K.F., Schneewind O. (1999). Purification and characterization of sortase, the transpeptidase that cleaves surface proteins of *Staphylococcus aureus* at the LPXTG motif. Proc. Natl. Acad. Sci. USA.

[17] Escaich S. (2008). Antivirulence as a new antibacterial approach for chemotherapy. Curr. Opin. Chem. Biol..

[18] Wang J., Qiu J., Tan W., Zhang Y., Wang H., Zhou X., Liu S., Feng H., Li W., Niu X. (2014). Fisetin inhibits *Listeria monocytogenes* virulence by interfering with the oligomerization of listeriolysin O. J. Infect. Dis..

[19] Cascioferro S., Totsika M., Schillaci D. (2014). Sortase A: An ideal target for anti-virulence drug development. Microb. Pathog..

[20] Macia M.D., Rojo-Molinero E., Oliver A. (2014). Antimicrobial susceptibility testing in biofilm-growing bacteria. Clin. Microbiol. Infect..

[21] Wang J., Zhou X., Liu S., Li G., Zhang B., Deng X., Niu X. (2015). Novel inhibitor discovery and the conformational analysis of inhibitors of listeriolysin O via protein-ligand modeling. Sci. Rep..

